# Analysis of Energy Harvesting Enhancement in Piezoelectric Unimorph Cantilevers

**DOI:** 10.3390/s21248463

**Published:** 2021-12-18

**Authors:** Mohammad Rahimzadeh, Hamid Samadi, Nikta Shams Mohammadi

**Affiliations:** 1 Department of Mechanical Engineering, Faculty of Engineering, Golestan University, Gorgan 4913815759, Iran; 2 Department of Mechanical Engineering, Babol University of Technology, Babol 4714873113, Iran; h.samadi99@stu.gu.ac.ir; 3 Department of Electrical Engineering, Shahrood University of Technology, Shahrood 3619995161, Iran; n.shamsmohammadi99@stu.gu.ac.ir

**Keywords:** piezoelectric, energy harvesting, unimorph, cantilever beam, resonant frequency

## Abstract

Environmental energy harvesting is a major operation in research and industries. Currently, researchers have started analyzing small-scale energy scavengers for the supply of energy in low-power electrical appliances. One area of interest is the use of piezoelectric materials, especially in the presence of mechanical vibrations. This study analyzed a unimorph cantilever beam in different modes by evaluating the effects of various parameters, such as geometry, piezoelectric material, lengths of layers, and the proof mass to the energy harvesting process. The finite element method was employed for analysis. The proposed model was designed and simulated in COMSOL Multiphysics, and the output parameters, i.e., natural frequencies and the output voltage, were then evaluated. The results suggested a considerable effect of geometrical and physical parameters on the energy harvesters and could lead to designing devices with a higher functional efficiency.

## 1. Introduction

Due to ever-increasing environmental concerns and the use of self-driven devices in pervasive wireless systems, harvesting energy from various environmental sources has received a great deal of attention [1]. There are different environmental energy sources such as solar energy [2], mechanical vibrations [3], heat [4], fluid flow [5], human body motions [6], and electromagnetic fields [7] that can be utilized for energy harvesting purposes. Energy harvesting from mechanical vibrations based on piezoelectric materials [8] is among the most convenient and attractive techniques for feeding small-scale devices.

The piezoelectric effect is defined as a linear-electromechanical reaction. Piezoelectrics are the materials on which electric charges appear during compression or tension [9]. Due to their ability to directly convert strain energy into useful electrical energy and their ease of use, these materials have been analyzed by many researchers [10]. When a poled piezoelectric material is strained, it becomes electrically polarized and produces an electric charge on its surface that can eventually be used in electronic devices [11].

Energy harvesting technology is now employed in many industries. An important area of use is with the Internet of Things (IoT), which aims to develop an ecosystem of different devices and establish comprehensive communications between smart devices, sensors, and simple actuators [12]. With advances in processor downsizing and the reduction in power consumption, the widespread deployment of actuators and sensors has become possible everywhere [13,14]. However, such an evolution needs a basic breakthrough in software and hardware development and data analysis. Because of the common usage of IoT devices installed in hard-accessible areas, their maintenance and regular battery replacement are impossible. Therefore, collecting and harvesting energy from ambient vibrations and providing sufficient energy for different devices can be a suitable solution. This process can significantly improve the lifetime of a device and eliminate the need for batteries used as an energy source [15].

In most cases, the structure of an energy scavenger is a cantilever beam with a piezoelectric layer [16,17]. Because of their relatively low resonance frequency and relatively high strain average per specific loading, cantilever beams are considered important [18]. If the beam has one piezoelectric layer, it is called unimorph [19]; however, if it has two piezoelectric layers, it is called bimorph [20]. Sometimes, a proof mass is also used at the free ends of the beams [21]. Due to frequency shift and strain distribution changes throughout the device, the proof mass affects the function of energy harvesters [22]. An important aspect of a piezoelectric energy harvesting system is the efficiency of the harvesting process and performance [23]. Recent studies aimed to reduce the natural frequency of systems to increase the application scope of environmental vibrations for energy harvesting [24]. As a result, a higher value of energy production can be reached even in environments with lower excitation values.

A variety of solutions were carried out to design devices whose natural frequency can be regulated based on the environment’s excitement frequency. One of these solutions is to apply axial tensile force with magnetic induction [25]. Other factors affecting the conversion efficiency of the energy harvester are electrically induced damping and AC/DC power output [26]. In addition, the invention of solutions to adjust the internal electrical impedance applied to the resistance load increases the efficiency of the energy harvester [27]. Applying nonlinear forces in linear vibration can help to increase the performance of these devices. Therefore, designing multi-stable systems such as bi-, tri-, and quad-stable systems attracted researchers’ attention [28].

Recent studies analyzed various geometries from simple beams including rectangular, trapezoidal, and triangular beams [29] to new geometries such as zigzag, sinusoidal, and spiral beams [30]. The variety of studied designs provided greater flexibility in application, resulted in different amounts of harvested energy, and, ultimately, improved the output power [31]. Changing the beam width was proposed as an appropriate strategy for increasing the output voltage and the power of energy scavengers. Researchers analyzed the output powers of several designs of cantilever beams and demonstrated the benefits of balancing strain distribution along the beam. According to their results, trapezoidal geometries have a higher efficiency compared to rectangular designs or the T-beams that have lower frequencies [32]. In a triangular beam, the output voltage increases due to uniform strain, as well as allowing for an increase in the strain average up to twice that of a similar load in comparison to a rectangular beam.

The effects of the length, thickness, and width of the piezoelectric layer were also analyzed in the literature, which indicated that the natural frequency of the triangular beam was the highest for all the considered parameters due to the mechanical properties of the beams. According to the results, the thickness of the harvesting beam’s layers affected the energy harvesting performance [33]. The homogenization of the distributions of axial strains along the beams improved the output power of energy harvesting. A common method of minimizing axial strain variation is to utilize beams with triangular or trapezoidal profiles [34,35].

In addition to considering the geometry of the harvesting beam, the effect of adding a proof mass needs to be taken into account. Adding the proof mass to the energy harvester beam can improve system performance and reduce the frequency ranges of energy harvesters. According to the results, the size, volume, and material of the proof mass had no considerable effect on the resonance frequency; however, its shape and position affected the output power by up to 2% and affected the resonance frequency by more than 10%. In addition, when the piezoelectric piece was positioned at a zero distance from the fixed end of the beam, it provided more electrical power due to the increase of mechanical strains than when it was positioned at its free end [36].

The research literature indicates that an attempt was made to increase the amount of extracted energy by providing practical solutions and reducing the resonant frequency of the system. We investigated the effectiveness of a broad spectrum of geometrical properties on the function of an energy harvester, which included the triangular, trapezius, and multi-steps together with parameters such as the ratio of the length of the piezoelectric layer to the substrate layer, the position of the piezoelectric patch, the effect of increasing the proof mass, and the properties of piezoelectric material. The simultaneous investigation of these parameters can lead to insight concerning the proper design of piezoelectric energy harvesters.

## 2. Electromechanical Model of the Piezoelectric Energy Scavenger

**Figure 1:**
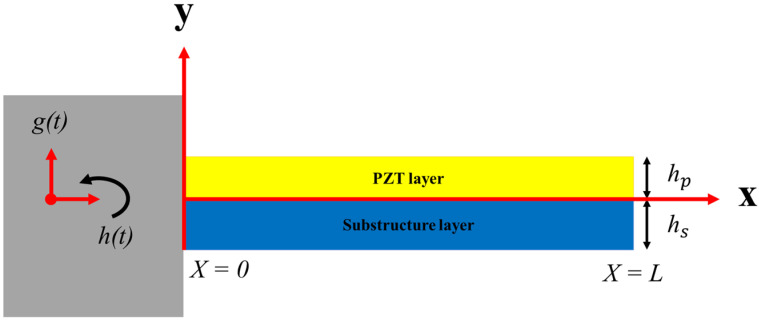
The schematic view of a unimorph cantilever beam.

Figure 1 demonstrates a cantilever beam with piezoelectric and substrate layers, which is a common model of energy harvesting.

Consider a beam with a width of b and a length of L that consists of an elastic layer with a thickness of $h_{s}$ and a piezoelectric layer with a thickness of $h_{p}$ as an energy harvester layer. Additionally, assume that the electrodes completely cover the surface of the piezoelectric layer and that the piezoelectric layer does not slip relative to the beam. Equation (1) describes the general motion of the beam under forced vibrations, including the motion of the beam base and its transverse displacements [37]:

(1) $$w\left(x,t\right)=w_{b}\left(x,t\right)+w_{r}\left(x,t\right)$$

where $w_{b}\left(x,t\right)$ is the movement of the beam base and $w_{r}\left(x,t\right)$ is the transverse displacement of the scavenger related to the clamped end. The movement of the beam base is defined in Equation (2):

(2) $$w_{b}\left(x,t\right)=g\left(t\right)+xh\left(t\right)$$

where g(t) is the translational motion of the beam on the *Y*-axis, and h(t)  is the rotation of the beam around the *Z*-axis. The equation of the beam’s plane vibrations is written as Equation (3) using the Euler–Bernoulli beam theory. Moreover, two different kinds of damping (i.e., external and internal damping) can be considered in the system to show mechanical losses [38].

(3) $$\frac{\partial^{2}M\left(x,t\right)}{\partial x^{2}}+C_{s}I\frac{\partial^{5}w_{rel}\left(x,t\right)}{\partial x^{4}\partial t}+C_{a}\frac{\partial w_{rel}\left(x,t\right)}{\partial t}+m\frac{\partial^{2}w_{rel}\left(x,t\right)}{\partial t^{2}}\;=-m\frac{\partial^{2}w_{b}\left(x,t\right)}{\partial t^{2}}-C_{a}\frac{\partial w_{b}\left(x,t\right)}{\partial t}$$

Accordingly, $C_{a}$ is the viscous air damping coefficient, $C_{s}$ is the equivalent coefficient of strain rate damping, I is the area moment of inertia, and m represents the linear mass density. The bending moment M(x,t) can be calculated through Equation (4) by using piezoelectric constitutive relationships.

(4) $$M\left(x,t\right)=-\int_{ha}^{hb}T_{1}^{s}by\;dy-\int_{hb}^{hc}T_{1}^{p}by\;dy$$

where b denotes the beam width, $T_{1}^{s}$ indicates the stress in the elastic layer, and $T_{1}^{p}$ refers to the stress in the piezoelectric layer. They are all obtained from Equations (5) and (6):

(5) $$T_{1}^{s}=Y_{s}S_{1}^{s}$$

(6) $$T_{1}^{p}=Y_{p}\left(S_{1}^{p}-d_{31}E_{3}\right)$$

where $d_{31}$ denotes the piezoelectric constant, $E_{3}$ indicates the electrical field, $S_{1}^{s}$ refers to strain at the elastic layer, $S_{1}^{p}$ represents the strain at the piezoelectric layer, and $Y_{p}$ and $Y_{s}$ are the Young’s modulus of the piezoelectric and substrate layers. The following equations result from the substitution of these variables:

(7) $$M\left(x,t\right)=\int_{ha}^{hb}Y_{s}b\frac{\partial^{2}w_{rel}\left(x,t\right)}{\partial x^{2}}y^{2}\;dy+\int_{hb}^{hc}Y_{p}b\frac{\partial^{2}w_{rel}\left(x,t\right)}{\partial x^{2}}y^{2}\;dy\;-\int_{hb}^{hc}v\left(t\right)Y_{p}b\frac{d_{31}}{h_{p}}y\;dy$$

(8) $$M\left(x,t\right)=YI\frac{\partial^{2}w_{rel}\left(x,t\right)}{\partial x^{2}}+\vartheta v\left(t\right)$$

in which $h_{a}$ is the lower position of the substrate layer, $h_{b}$ is the higher position of the substrate, and $h_{c}$ is the higher position of the piezoelectric layer from the neutral axis. Additionally, v(t) is the voltage value of two ends of the piezoelectric layer. The bending stiffness of the composite beam and the electromechanical couplings of the structure can be expressed by the following equations:

(9) $$YI=b\left[\frac{Y_{s}\left(h_{b}^{3}-h_{a}^{3}\right)+Y_{p}\left(h_{c}^{3}-h_{b}^{3}\right)}{3}\right]$$

(10) $$\vartheta =-\frac{Y_{p}d_{31}b}{2h_{p}}\left(h_{c}^{2}-h_{b}^{2}\right)$$

(11) $$M\left(x,t\right)=YI\frac{\partial^{2}w_{rel}\left(x,t\right)}{\partial x^{2}}+\vartheta v\left(t\right)\left[H\left(x\right)-H\left(x-L\right)\right]$$

where H(x) is the Heaviside equation. Equation (12) results from the substitution of the above equations into Equation (3):

(12) $$\begin{array}{l} YI\frac{\partial^{4}w_{rel}\left(x,t\right)}{\partial x^{4}}+C_{s}I\frac{\partial^{5}w_{rel}\left(x,t\right)}{\partial x^{4}\partial t}+C_{a}\frac{\partial w_{rel}\left(x,t\right)}{\partial t}+m\frac{\partial^{2}w_{rel}\left(x,t\right)}{\partial t^{2}}\\ \;+\vartheta v\left(t\right)\left[\frac{d\delta \left(x\right)}{dx}-\frac{d\delta \left(x-L\right)}{dx}\right]=-m\frac{\partial^{2}w_{b}\left(x,t\right)}{\partial t^{2}}-C_{a}\frac{\partial w_{b}\left(x,t\right)}{\partial t} \end{array}$$

where δ(x) is the Dirac delta function. To obtain the equation for the electrical and mechanical fields, it is necessary to define the piezoelectric constitutive relationship, which is expressed as the following equations:

(13) $$D_{3}=d_{31}T_{1}+\varepsilon_{33}^{T}E_{3}$$

(14) $$D_{3}\left(x,t\right)=d_{31}Y_{p}S_{1}\left(x,t\right)+\varepsilon_{33}^{T}\frac{v\left(t\right)}{h_{p}}$$

(15) $$S_{1}\left(x,t\right)=-h_{pc}\frac{\partial^{2}w_{rel}\left(x,t\right)}{\partial x^{2}}$$

(16) $$D_{3}\left(x,t\right)=-d_{31}Y_{p}h_{pc}\frac{\partial^{2}w_{rel}\left(x,t\right)}{\partial x^{2}}-\varepsilon_{33}^{T}\frac{v\left(t\right)}{h_{p}}$$

where $h_{pc}$ indicates the distance between the neutral axis and the center of the piezoelectric layer, $D_{3}$ denotes the electrical displacement, and $\varepsilon_{33}^{T}$ indicates permittivity, whereas $E_{3}$ refers to the electric field. The values of current i(t) and voltage v(t) can be calculated with respect to the electric charge q(t) through the following equations [37]:

(17) $$q\left(t\right)=\int_{A}^{}D.ndA=-\int_{\mathrm{x=0}}^{L}\left(d_{31}Y_{p}h_{pc}\frac{\partial^{2}w_{rel}\left(x,t\right)}{\partial x^{2}}+\varepsilon_{33}^{T}\frac{v\left(t\right)}{h_{p}}\right)dx$$

(18) $$i\left(t\right)=\frac{dq\left(t\right)}{dt}=-\int_{\mathrm{x=0}}^{L}d_{31}Y_{p}h_{pc}b\frac{\partial^{3}w_{rel}\left(x,t\right)}{\partial x^{2}\partial t}dx-\frac{\varepsilon_{33}^{T}bL}{h_{p}}\frac{dv\left(t\right)}{dt}$$

(19) $$v\left(t\right)=R_{l}i\left(t\right)=-R_{l}\left[\int_{\mathrm{x=0}}^{L}d_{31}Y_{p}h_{pc}b\frac{\partial^{3}w_{rel}\left(x,t\right)}{\partial x^{2}\partial t}dx-\frac{\varepsilon_{33}^{T}bLdv\left(t\right)}{h_{p}dt}\right]\frac{\varepsilon_{33}^{T}bLdv\left(t\right)}{h_{p}dt}\;+\frac{v\left(t\right)}{R_{l}}=-\int_{\mathrm{x=0}}^{L}d_{31}Y_{p}h_{pc}b\frac{\partial^{3}w_{rel}\left(x,t\right)}{\partial x^{2}\partial t}dx$$

where $R_{l}$ denotes the electrical resistance of the circuit. D is the vector of electric displacements and n is the unit outward normal. The transverse displacement of the beam can be defined as Equation (20) to solve the governing equations:

(20) $$w_{rel}\left(x,t\right)=\overset{n}{\underset{r=1}{\sum }}\emptyset_{r}\left(x\right)\;\eta_{r}\left(t\right)$$

where $\emptyset_{r}\left(x\right)$ represents the normalized eigenfunction and $\eta_{r}$(*t*) denotes the modal coordinate of the cantilever beam.

(21) $$\frac{dv\left(t\right)}{dt}+\frac{h_{p}}{\varepsilon_{33}^{s}bLR_{l}}v\left(t\right)=\overset{\infty }{\underset{r=1}{\sum }}\varphi_{r}\frac{d\eta_{r}\left(x\right)}{dt}$$

By multiplying the integral factor $\mu =e^{\frac{-t}{\tau_{c}}}$, the equation can be solved and rewritten as Equation (22):

(22) $$v\left(t\right)=e^{\frac{-t}{\tau_{c}}}\left[\overset{\infty }{\underset{r=1}{\sum }}\varphi_{r}\int^{\;}e^{\frac{t}{\tau_{c}}}\frac{d\eta_{r}\left(x\right)}{dt}dt\right]$$

where

(23) $$\varphi_{r}=-\frac{d_{31}Y_{p}h_{pc}h_{p}}{\varepsilon_{33}^{s}L}\int_{\mathrm{x=0}}^{L}\frac{d^{2}\emptyset_{r}\left(x\right)}{dx^{2}}dx=-\frac{d_{31}Y_{p}h_{pc}h_{p}}{\varepsilon_{33}^{s}L}\frac{d\emptyset_{r}\left(x\right)}{dx}|\begin{array}{c} x=L \end{array}$$

and

(24) $$\emptyset_{r}\left(x\right)=\sqrt{\frac{1}{mL}}\left[\cosh\frac{\lambda_{r}}{L}x-\cos\frac{\lambda_{r}}{L}x-\sigma_{r}\left(\sinh\frac{\lambda_{r}}{L}x-\sin\frac{\lambda_{r}}{L}x\right)\right]$$

where $\lambda_{r}$ indicates the dimensionless eigenvalues and can be specified by solving the characteristic equations

(25) 1+cosλcoshλ=0

(26) $$\sigma_{r}=\frac{\sinh\lambda_{r}-\sin\lambda_{r}}{\cosh\lambda_{r}+\cos\lambda_{r}}$$

Moreover, $\omega_{r}$ denotes the natural frequency of the system obtained from the following equation:

(27) $$\omega_{r}=\lambda_{r}^{2}\sqrt{\frac{YI}{mL^{4}}}$$

The modal mechanical response is obtained from the following equations by solving Equation (27):

(28) $$\eta_{r}\left(x\right)=\frac{\left[m\left(x\right)\omega^{2}\left(\gamma_{r}^{w}Y_{0}+\gamma_{r}^{\theta }\theta_{0}\right)-\chi_{r}V_{0}e^{j\omega t}\right]}{\omega_{r}^{2}-\omega^{2}+j2\zeta_{r}\omega_{r}\omega }$$

(29) $$\gamma_{r}^{w}=\int_{0}^{L}\emptyset_{r}\left(x\right)dx$$

(30) $$\gamma_{r}^{\theta }=\int_{0}^{L}x\emptyset_{r}\left(x\right)dx$$

It is also supposed that:

(31) $$h\left(t\right)=\theta_{0}e^{j\omega t}$$

(32) $$g\left(t\right)=Y_{0}e^{j\omega t}$$

(33) $$v\left(t\right)=V_{0}e^{j\omega t}$$

Therefore,

(34) $$v\left(t\right)=\frac{\sum_{r=1}^{\infty }\frac{-jm\omega \varphi_{r}\left(\gamma_{r}^{w}Y_{0}+\gamma_{r}^{\theta }\theta_{0}\right)e^{j\omega t}}{\omega_{r}^{2}-\omega^{2}+j2\zeta_{r}\omega_{r}\omega }}{\sum_{r=1}^{\infty }\frac{j\omega \chi_{r}\varphi_{r}}{\omega_{r}^{2}-\omega^{2}+j2\zeta_{r}\omega_{r}\omega }+\frac{1+j\omega \tau_{c}}{\tau_{c}}}$$

By substituting these values, the voltage will be defined as [37]:

(35) $$\frac{v\left(t\right)}{-\omega^{2}Y_{0}e^{j\omega t}}=\frac{\sum_{r=1}^{\infty }\frac{-jm\omega \varphi_{r}\gamma_{r}^{w}}{\omega_{r}^{2}-\omega^{2}+j2\zeta_{r}\omega_{r}\omega }}{\sum_{r=1}^{\infty }\frac{j\omega \chi_{r}\varphi_{r}}{\omega_{r}^{2}-\omega^{2}+j2\zeta_{r}\omega_{r}\omega }+\frac{1+j\omega \tau_{c}}{\tau_{c}}}$$

## 3. Problem Statement

Table 1 presents the geometrical and mechanical characteristics of the substrate layer, the piezoelectric patch, and the proof mass. It should be noted that the beams were fixed from the smaller side of the base. This study aimed to analyze the strategies for improving the energy efficiency in a unimorph cantilever beam and presents solutions related to the geometrical and physical properties of cantilever beams.

The effect of disparate factors on energy harvester performance were examined in several parts. First, a comparative study was carried out using various piezoelectric materials. Beam shape was assumed to be trapezius and the size of the layers was assumed to be the same. Five materials, aluminum nitride, lead zirconate titanate (PZT-5H), barium sodium niobate, lithium niobate, and lithium tantalate, were employed in simulations as piezoelectric layers and their results were compared.

**Figure 2:**
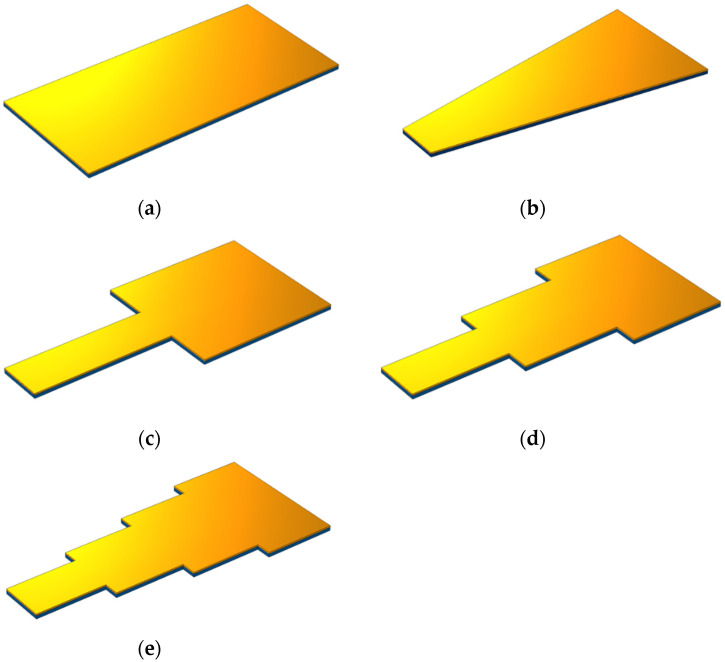
Different geometries of a cantilever beam: (**a**) rectangular, (**b**) trapezoidal, (**c**) two-step, (**d**) three-step, and (**e**) four-step geometries.

In the next part, five rectangular, trapezius, two-step, three-step, and four-step beams were compared to study the role of device geometry on the value of extracted energy (Figure 2). The resonance frequency of the vibrating beam as well as the output voltage obtained from the base vibration were investigated.

To examine the effect of the ratio of the piezoelectric layer length to the substrate layer length, four disparate ratios were studied in the next part. As the length of the piezoelectric layer is represented by $L_{p}$ and the length of the substrate layer is represented by $L_{s}$, the study was carried out in the following length ratios:

(36) $$\;\frac{L_{p}}{L_{s}}=\frac{1}{2},\;\frac{1}{4},\;\frac{1}{6},\;\frac{1}{8}$$

**Figure 3:**
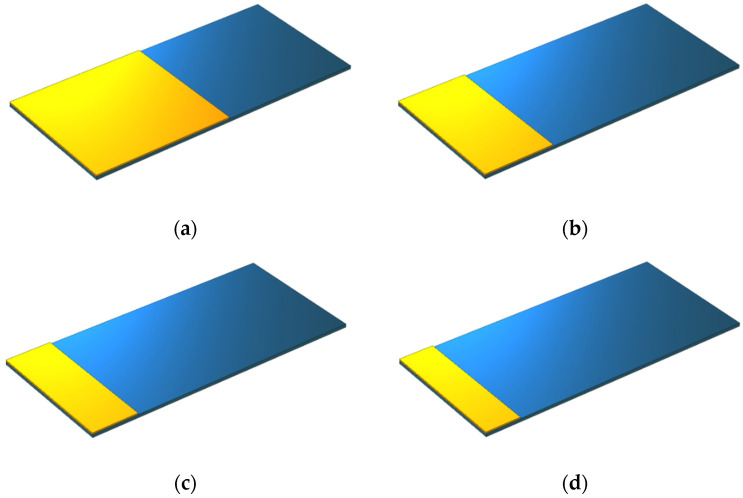
The schematic view of a geometric model of a rectangular beam while changing the length of the piezoelectric patch relative to that of the substrate layer: (**a**) LpLs=12, (**b**)  LpLs=14, (**c**)  LpLs=16, and (**d**)  LpLs=18.

In all states, the beginning of the piezoelectric layer was assumed to be at a zero distance from the fixed section of the beam (Figure 3).

**Figure 4:**
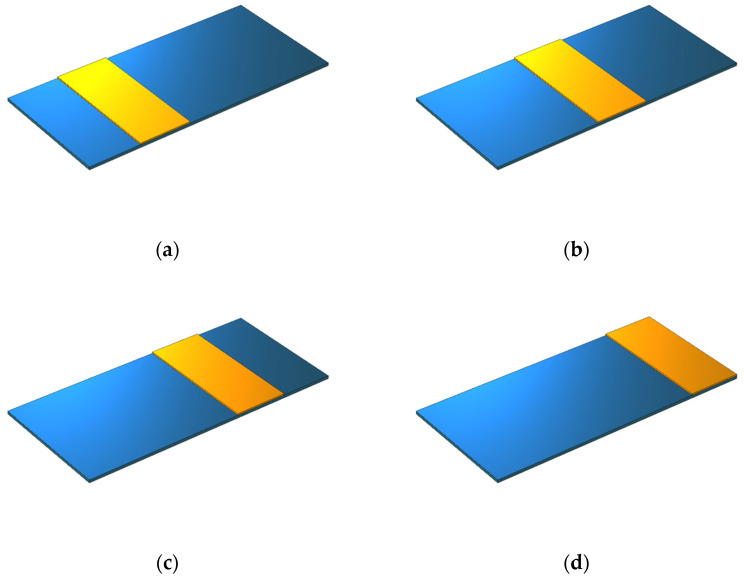
The geometric model of a rectangular beam while changing the position of the piezoelectric patch: (**a**) $x_{0p}=15\;\mathrm{mm}$, (**b**) $x_{0p}=30\;\mathrm{mm}$, (**c**) $x_{0p}=45\;\mathrm{mm}$, and (**d**) $x_{0p}=60\;\mathrm{mm}$.

In another study, the piezoelectric patch length was regarded as fixed; thus, the impact of the position of this layer on the performance of the device was assessed. Therefore, the length of the piezoelectric layer was assumed to be one-fifth of the substrate layer ($L_{p}/L_{s}=1/5$). The piezoelectric layer was simulated in four states and at distances of 15, 30, 45, and 60 mm from the fixed section of the beam (Figure 4). The output voltage amounts were extracted in terms of the excitation frequency.

The effect of positioning the proof mass at the end of the free beam was studied in the subsequent part. The effective parameters, such as output voltage, resonance frequency value, and the mode shapes at two states, were compared, both with and without the proof mass.

**Figure 5:**
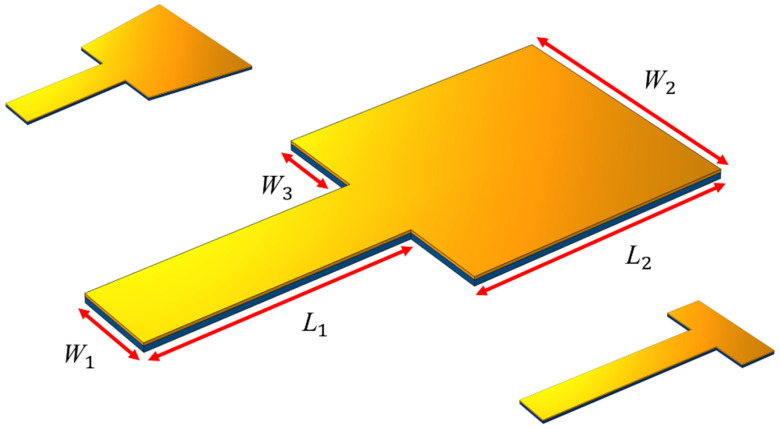
The schematic view of the studied two-step beam.

Eventually, per the results from the previous parts, one two-step cantilever beam was selected for a more precise study (Figure 5). Three different states were considered: First, all geometrical properties were assumed to be constant, and the effect of the change of the width of the free end of the beam ($W_{2}$) was studied. Then, the lengths of the steps ($L_{1}=L_{2}$) were assumed to be the same, and the effect of the change in the overall length of the device on the output voltage was examined. Finally, the total length of the device ($L_{1}+L_{2}=cte.$) was assumed to be fixed, and the effect of the changes in the length of each step on the performance of the device was investigated.

**Figure 6:**
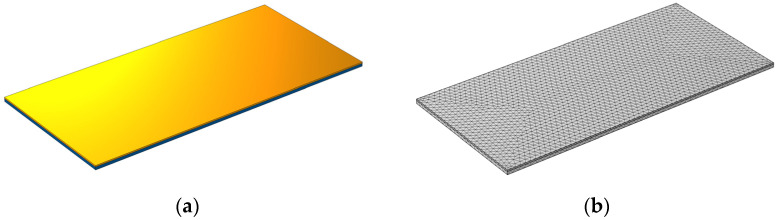
The geometrical model of the rectangular beam: (**a**) geometrical model in COMSOL Multiphysics and (**b**) meshed model.

Beam geometry modeling and simulations were performed in COMSOL Multiphysics software (COMSOL Inc, Los Angeles, CA, USA). Moreover, meshing of fine quality was implemented automatically in all models (Figure 6). Tetrahedral elements were used in the simulation. Additionally, the vibration of the beam’s base was applied as a 0.1 *g* body force, and the damping coefficient was fixed in the total frequency interval and assumed to be 0.01.

## 4. Results and Discussion

**Figure 7:**
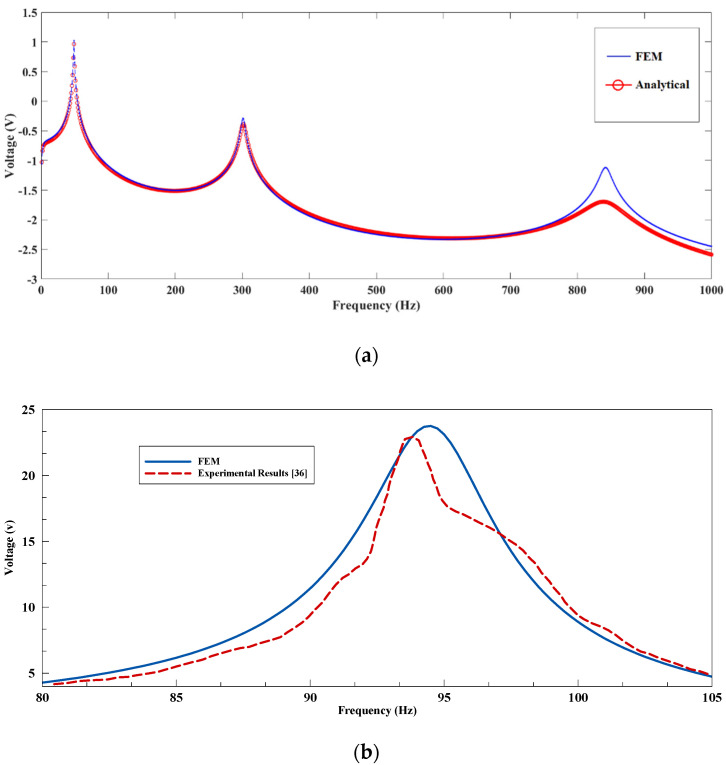
The comparison of the COMSOL Multiphysics simulation results (**a**) in logarithmic scale with the analytical results (Equation (35)) within the range of 0 to 1000 Hz for $R_{l}=10^{6}\;\Omega$ and (**b**) with the experimental results reported by Pradeesh and Udhayakumar within the range of 80 to 105 Hz [36].

To ensure the accuracy of the simulation trend from COMSOL Multiphysics software, its results were validated through two analytical and experimental results: first, Equation (35) was used in an analytical approach, the output voltage diagram was obtained for the rectangular cantilever beam, and the resistive load of $10^{6}\;\Omega$ was obtained using MATLAB software. The experimental results matched the results of the COMSOL simulation (Table 2 and Figure 7a). The lack of adjustment of the two diagrams at high frequencies was due to the different damping ratio for higher vibrational modes at the analytical level, which were assumed to be constant in the COMSOL simulation. Validation of the simulation results with reported experimental data [36] was also performed. The results of the simulation properly matched the experimental results, which emphasized the accuracy of the modeling process (Figure 7b). The current diagrams can be acquired by dividing the amount of voltage by the resistive load; thus, it has a trend similar to the voltage diagram.

**Figure 8:**
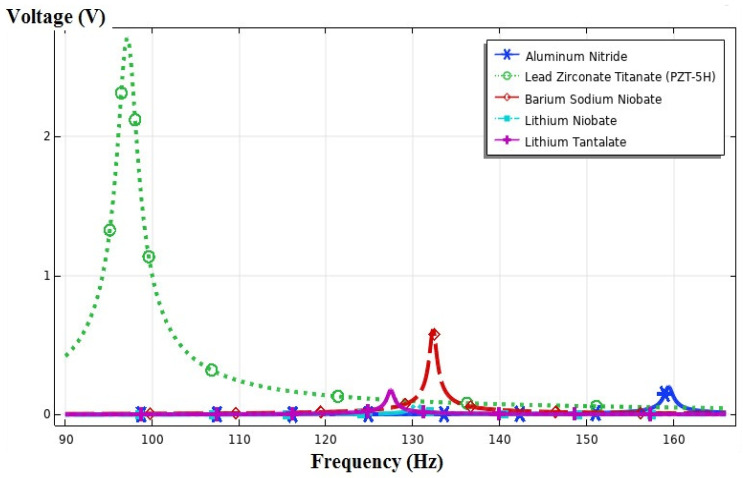
The output voltage vs. the frequency for five different piezoelectric materials.

Five different materials were applied to the piezoelectric layer of the trapezoidal beam. The material library of COMSOL Multiphysics software was used to apply the physical and mechanical properties of the piezoelectric layers, and the highest amount of harvested energy was observed in PZT-5H and barium sodium niobate. As seen in Figure 8, the highest amount of energy was harvested by changing the piezoelectric material to PZT-5H and minimizing the resonance frequency. The maximum output voltage at this state amounted to 2.05 V, which was created at 98.1 Hz. The minimum output voltage occurred when using lithium niobate, and it amounted to 0.078 V at the frequency of 130.4 Hz.

After examining the effect of disparate piezoelectric materials on the energy harvester’s efficiency, the function of the device was investigated using five different geometries, as per Figure 2. The piezoelectric layer was assumed to be PZT-5H in each shape. The first natural frequency and the maximum amount of energy harvested with each geometry are presented thoroughly in Table 3. The amount of harvested energy increased to 2.7 V by changing the geometry from a rectangle to a trapezoid. The first natural frequency also decreased significantly. However, the highest voltage of the geometries was observed at 3.3 V in the two-step geometry.

**Figure 9:**
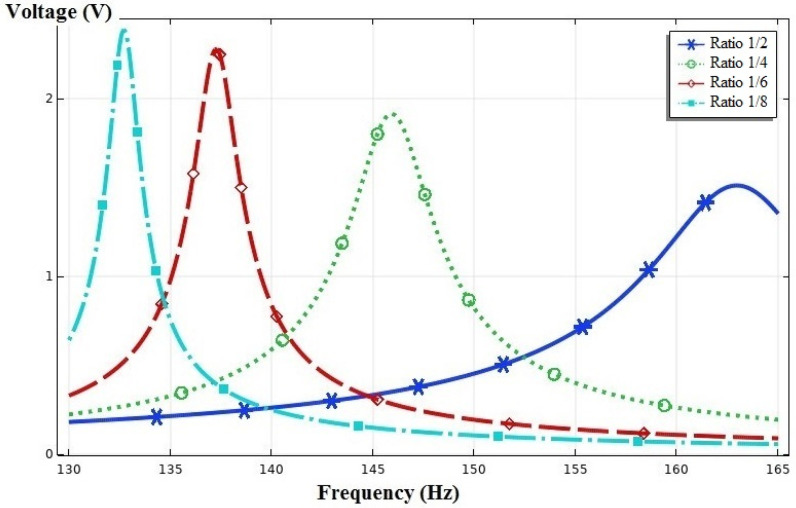
The output voltage vs. the frequency for $\frac{L_{p}}{L_{s}}=\frac{1}{2},\;\frac{1}{4},\;\frac{1}{6},\;\mathrm{and}\;\frac{1}{8}$.

The effect of the ratio of the length of the piezoelectric layer to the substrate layer was investigated according to Figure 3. The length of the substrate layer was fixed at 75 mm. According to Figure 9, the ratio of the length of the piezoelectric patch to the length of the substrate layer was analyzed in four cases of 1/2, 1/4, 1/6, and 1/8. According to the results, when the length of the piezoelectric layer was one-eighth of the substrate layer, the maximum output voltage was 2.3 V observed at a frequency of 133 Hz. The increase in the ratio of the length of the piezoelectric layer to the substrate layer from 1/8 to 1/2 resulted in the reduction in the output voltage to 1.5 V and the increase in the resonance frequency up to 163 Hz.

**Figure 10:**
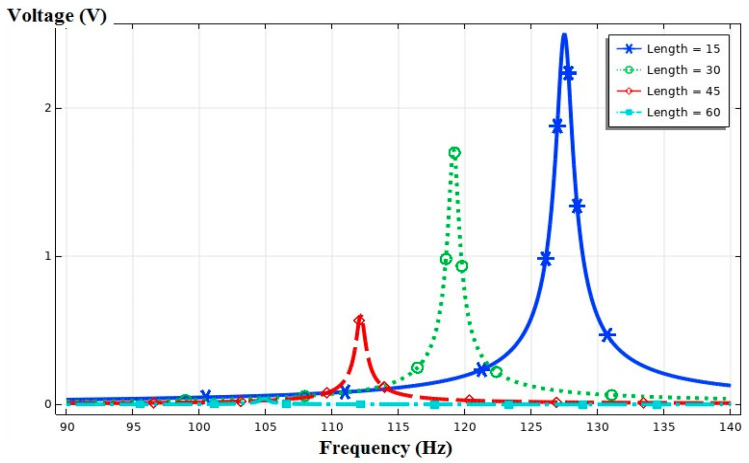
The output voltage vs. the frequency at different distances of the piezoelectric patch from the cantilevered end.

The voltage in each class could be calculated and compared by considering the length of the piezoelectric patch to be one-fifth of the length of the substrate layer and moving it from the beginning to the end of the beam (Figure 4). Figure 10 shows that the maximum output voltage among the studied items was in state (a). This voltage of 2.5 V created a resonance frequency of 127.5 Hz. When the piezoelectric patch was located at the free end of the beam, the voltage amount dropped to 0.04 V.

**Figure 11:**
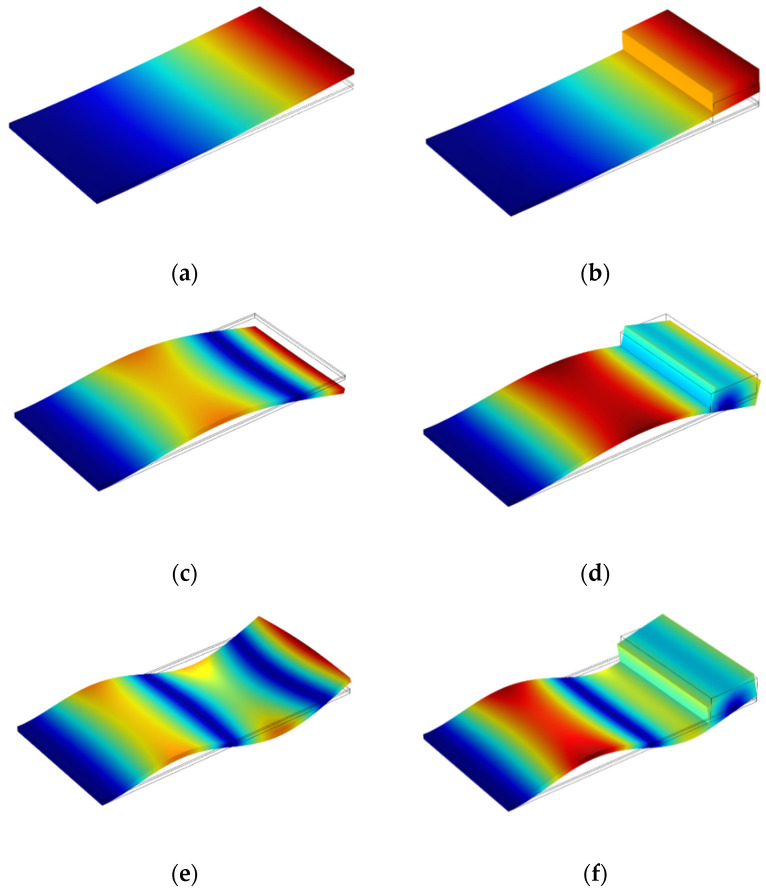
The mode shapes of the rectangular beam with and without a proof mass: (**a**,**b**) first mode shape, (**c**,**d**) second mode shape, and (**e**,**f**) third mode shape.

We aimed to reduce the first resonant frequency and increase the harvested energy. The initial natural frequency was reduced by 73 Hz by adding the proof mass to the end of the rectangular beam. The output voltage also increased from 2.25 to 4.35 V, which is equal to 93%. Figure 11 and Table 4 demonstrate the mode shapes and the values of the natural frequencies of the rectangular beam with and without the proof mass.

As per the results from the previous stages, the two-step geometry was evaluated in greater detail. The effects of the represented parameters are investigated in Figure 5 at three states: first, all dimensions were assumed to be constant; second, the width of the free end ($W_{2}$) was changed from 12 to 48 mm, and, finally, its effect was studied on the extracted energy and resonance frequency. As seen in Table 5, the highest level of harvested energy was related to the state $W_{2}=48$, which was 3.76 V.

**Figure 12:**
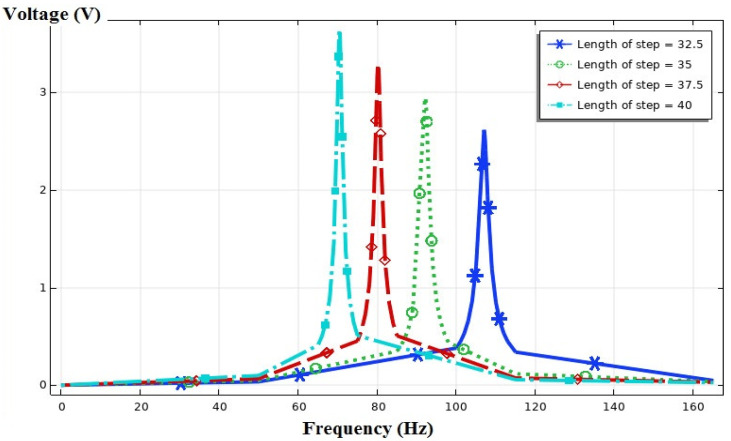
The output voltage vs. the frequency for different two-step beam lengths, $L_{1}=L_{2}=\left[32.5,\;35,\;37.5,\;40\right]\;\mathrm{mm}$.

At the next stage, each width of the beam was assumed to be constant. The length of the beam was changed such that the length of the two steps became equal ($L_{1}=L_{2}$). Figure 12 shows four states concerning the different lengths of the two-step beam. As per Figure 12, the maximum output voltage occurs when the length of the beam is at its longest (80 mm), and the maximum voltage at this state amounts to 3.6 V.

At the final stage, the length of the beam was assumed to be 75 mm and the length of each step was changed such that the overall length could remain fixed ($L_{1}+L_{2}=cte$). As seen in Table 6, the highest levels of harvested energy were related to $L_{2}=50$ and $L_{1}=25$ mm at 3.5 V.

## 5. Conclusions

This study simulated and analyzed five different geometries of piezoelectric unimorph cantilevers: rectangular, trapezoidal, two-step, three-step, and four-step. Different analyses of each geometry were performed parametrically using COMSOL Multiphysics software. The results were then compared. PZT-5H had the highest output voltage, which was found by selecting five different piezoelectric materials in the trapezoidal geometry. Barium sodium niobate and aluminum nitrate had the second and third highest output voltages, respectively.

The frequency domain analysis indicated that the rectangular geometry had the lowest value, whereas the two-step geometry had the highest output voltage of all the geometries. Moreover, increasing the number of beam steps had an inverse relationship with the amount of harvested energy. The trapezoidal geometry simulation showed that the output voltage increased. Therefore, in the two-step geometry, the geometry of the second step was changed from rectangular to trapezoidal, which increased the output voltage by 15% at most.

The length of the beam affected various values, including the natural frequency value and the output voltage. According to the analysis of variations in the length of a two-step beam when the length of the substrate layer and that of the piezoelectric were the same, increasing the total length of the beam increased the amount of energy absorption. Furthermore, the considered beam was analyzed where the total length of the beam was constant with the length of each step being changed; therefore, the rates of the improved voltage were up to 7% and 56% compared with the steps of equal length in two-step geometry and the rectangular geometry, respectively.

In the next phase, the effects of changing the length were analyzed. Comparing the length ratio of the layers indicated that a higher output voltage was obtained when the length of the piezoelectric patch was one-eighth of the substrate layer. Next, the amount of the output voltage was compared when the substrate layer was five times longer than the piezoelectric patch. In this case, the piezoelectric patch was placed at different distances from the clamped end of the beam. Moreover, it had the highest output voltage when the piezoelectric piece was attached to the clamped end. Adding a proof mass to the end of the rectangular beam and comparing it with a simple beam also resulted in a 93% increase in the energy uptake. Furthermore, the addition of the proof mass reduced the natural frequency, a strategy that can be adopted to bring the natural frequency closer to the frequency of the ambient vibrations.

## Figures and Tables

**Table 1:** The geometrical and mechanical characteristics of the substrate layer, piezoelectric patch, and proof mass.

Parameter	Steel	PZT-5H	Proof Mass
Young’s Modulus (GPa)	200	67	200
Density (kg/m^3^)	7850	7800	7850
Length (mm)	75	75	15
Width (mm)	36	36	36
Thickness (mm)	0.8	0.4	5
$\mathrm{Piezoelectric}\;\mathrm{constant},\;d_{31}$ (pm/V)	----	−190	----
$\mathrm{Permittivity},\;\varepsilon_{33}^{s}$ (nF/m)	----	15.93	----

**Table 2:** Comparing the values of the natural frequencies obtained from COMSOL and MATLAB software (The MathWorks, Natick, MA, USA).

	COMSOL	MATLAB
1st natural frequency (Hz)	47.82	47.81
2nd natural frequency (Hz)	299.65	299.61
3rd natural frequency (Hz)	838.81	838.90

**Table 3:** The amount of voltage harvested from various studied geometries.

Case	1st Natural Frequency (Hz)	Output Voltage (V)
a	137.9	2.2
b	97.1	2.7
c	80.5	3.3
d	84.3	3.2
e	86.6	3.0

**Table 4:** The natural frequencies for the rectangular beam with and without a proof mass.

Case	1st Natural Frequency (Hz)	2nd Natural Frequency (Hz)	3rd Natural Frequency (Hz)	Output Voltage (V)
Without proof mass	138	858.3	2406.4	2.25
With proof mass	73	700	2058	4.35

**Table 5:** The amount of energy harvested in the two-step beam. ($W_{1}=W_{3}=12$ mm and $L_{1}=L_{2}=32.5$ mm).

$W_{2}\;\left(\mathrm{mm}\right)$	1st Natural Frequency (Hz)	Output Voltage (V)
12	106.2	2.32
24	90.7	2.81
36	80.5	3.29
48	73.1	3.76

**Table 6:** The amount of energy harvested in the two-step beam. ($W_{1}=12,\;W_{2}=36,\;W_{3}=12\;\mathrm{mm}$).

$L_{1}\;\left(\mathrm{mm}\right)$	$L_{2}\;\left(\mathrm{mm}\right)$	1st Natural Frequency (Hz)	The Output Voltage (V)
12.5	62.5	90.9	3.43
25	50	82.4	3.5
37.5	37.5	80.5	3.29
50	25	83.4	2.85
62.5	12.5	94.3	2.21

## Data Availability

Not applicable.
